# miR-132 Mediates the Integration of Newborn Neurons into the Adult Dentate Gyrus

**DOI:** 10.1371/journal.pone.0019077

**Published:** 2011-05-17

**Authors:** Bryan W. Luikart, AeSoon L. Bensen, Eric K. Washburn, Julia V. Perederiy, Kimmy G. Su, Yun Li, Steven G. Kernie, Luis F. Parada, Gary L. Westbrook

**Affiliations:** 1 The Vollum Institute, Oregon Health and Science University, Portland, Oregon, United States of America; 2 Department of Developmental Biology and Kent Waldrep Foundation Center for Research on Nerve Growth and Regeneration, University of Texas Southwestern Medical School, Dallas, Texas, United States of America; The Research Center of Neurobiology-Neurophysiology of Marseille, France

## Abstract

Neuronal activity enhances the elaboration of newborn neurons as they integrate into the synaptic circuitry of the adult brain. The role microRNAs play in the transduction of neuronal activity into growth and synapse formation is largely unknown. MicroRNAs can influence the expression of hundreds of genes and thus could regulate gene assemblies during processes like activity-dependent integration. Here, we developed viral-based methods for the *in vivo* detection and manipulation of the activity-dependent microRNA, miR-132, in the mouse hippocampus. We find, using lentiviral and retroviral reporters of miR-132 activity, that miR-132 is expressed at the right place and right time to influence the integration of newborn neurons. Retroviral knockdown of miR-132 using a specific ‘sponge’ containing multiple target sequences impaired the integration of newborn neurons into the excitatory synaptic circuitry of the adult brain. To assess potential miR-132 targets, we used a whole-genome microarray in PC12 cells, which have been used as a model of neuronal differentiation. miR-132 knockdown in PC12 cells resulted in the increased expression of hundreds of genes. Functional grouping indicated that genes involved in inflammatory/immune signaling were the most enriched class of genes induced by miR-132 knockdown. The correlation of miR-132 knockdown to increased proinflammatory molecular expression may indicate a mechanistic link whereby miR-132 functions as an endogenous mediator of activity-dependent integration *in vivo*.

## Introduction

Newborn neurons are continuously born and functionally integrate into the adult dentate gyrus. This process has been implicated in diverse functions such as learning and mood regulation [1]. Enhanced neurogenesis in mice correlates with all known antidepressant treatments including exercise, medications, and electroconvulsive therapy [2]. Further, ablation of adult neurogenesis by irradiation or gene deletion prevents the normal behavioral response to antidepressant therapy [3], [4]. Neuronal activity elicited by stimuli ranging from enriched environment to seizures enhances growth and survival of newborn neurons [5], [6]. Thus neurogenesis and subsequent integration of new neurons into the adult circuitry serve as sensitive indicators of activity-dependent neural plasticity. Understanding the molecular mechanisms that govern the birth and maturation of newborn neurons in the adult may provide strategies to manipulate activity-dependent neural function for therapeutic gain.

As progenitor cells undergo the transition into differentiated neurons and integrate into the adult circuit, there are large-scale changes in gene expression [7]. Activity-dependent genes are ideal candidates for orchestrating this transition. For example, phosphorylation of cAMP response element binding protein (CREB) is increased in newborn neurons during integration [8], [9]. Likewise, antidepressants as well as seizures drive CREB activity and enhance the integration of newborn neurons in animal models [9], [10], [11]. This CREB activation regulates the maturation and survival of newborn neurons [12]. In defining the CREB regulon, several microRNAs appeared as potential CREB targets [13]. MicroRNAs are endogenously expressed ∼22 nucleotide RNAs, which downregulate the expression of large numbers of target genes *in vivo* [14], [15]. This novel class of molecules can potentially affect large-scale changes in the proteome of a cell to direct processes such as differentiation and maturation.

To address whether the CREB-regulated miR-132 [16], [17], [18] influences the functional integration of newborn neurons into the adult dentate gyrus *in vivo*, we developed a set of novel tools to examine its expression and function. As detected with a lentiviral reporter, miR-132 was first expressed after transient amplifying cells differentiate into neurons, and then increased further in mature granule cells. To knockdown miR-132 in newborn neurons, we developed a retroviral “sponge”, and then assessed synaptic function after 21 days using whole-cell recording. In neurons expressing the sponge, there was a decrease in dendritic spines and very little spontaneous excitatory activity indicating that miR-132 is necessary for robust excitatory synapse formation. Paired recordings from neighboring newborn neurons confirmed that miR-132 knockdown resulted in decreased integration into the perforant path circuit. Our results suggest that miR-132 plays a central role in the genetic program that drives activity-dependent integration of newborn neurons into the hippocampal circuitry. We also performed microarray experiments in PC12 cells to gain insight into the molecular changes that may mediate this phenotype.

## Results

### miR-132 is expressed in maturing neurons

**Figure 1:**
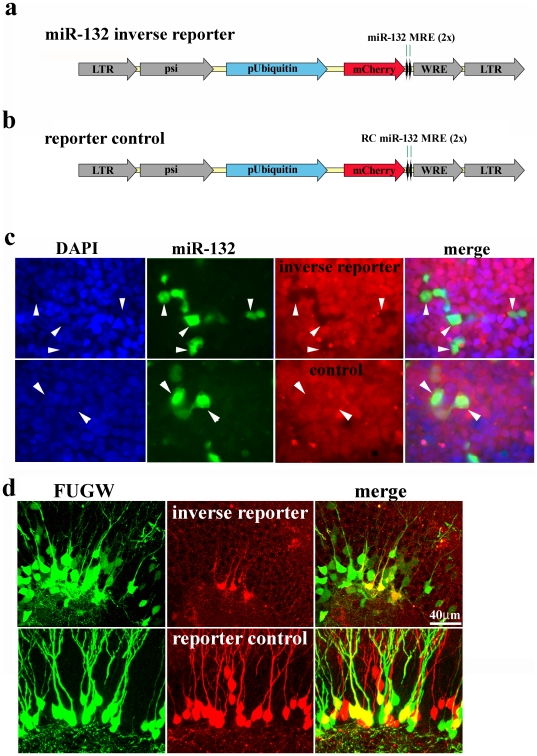
An Inverse Reporter for Detecting miR-132 Expression *In Vivo*. **a,** We generated an inverse miR-132 reporter lentivirus by placing two perfectly complementary miR-132 target sequences (miR-132 MRE) downstream of mCherry driven by an internal ubiquitin promoter (pUbiquitin). This inverse reporter design results in suppression of mCherry expression in the presence of miR-132. **b,** The reporter control virus was generated by placing the reverse complement of the miR-132 target (RC miR-132 MRE) downstream of mCherry. **c**, Hek293 cells were infected with either the inverse reporter or reporter control lentivirus. Single cells were cultivated to generate clonal cell lines expressing the miR-132 inverse reporter (top row) or the inverse reporter control (bottom row). Infection of a proportion of the cells with a miR-132-expressing retrovirus (green) suppressed mCherry in the inverse reporter cells (black silhouettes in red panel, top row), but not in inverse reporter control cells (red panel, bottom row). **d,** We co-injected equal titers of the GFP-expressing FUGW virus and either the mCherry-expressing miR-132 inverse reporter virus or the reporter control virus into 8 week old mice. FUGW lentivirus showed widespread infection of granule cells at 7 days post-injection (DPI, left panels). In contrast, the mCherry expressing miR-132 inverse reporter virus primarily labeled cells along the subgranular zone of the dentate gyrus (top row, middle panel). The merge is shown in the right panel. Infection of the reporter control virus showed the same widespread infection as the GFP-expressing FUGW virus (lower row, left and middle panel).

MicroRNAs act by decreasing expression of or degrading messenger RNAs. Thus a reporter carrying specific microRNA target sequences would be expected to decrease its expression when the microRNA activity increases, i.e. an “inverse” reporter. We developed such an inverse fluorescent reporter to track miR-132 expression *in vivo* (Fig. 1). We generated a miR-132 inverse reporter by cloning two perfect miR-132 target sites in the 3′ UTR of mCherry in a lentiviral vector (Fig. 1a). As a reporter control we cloned the reverse complement of the miR-132 targets into the mCherry UTR (Fig. 1b). The resulting vectors were packaged to generate high titer viral particles. The mature miR-132 sequence is 100% conserved in mice, rats, and humans. We could therefore evaluate our reagents in a variety of *in vitro* systems. To assess the validity of the approach, HEK293 (ATCC) cells were infected with these viruses, and individual cells were isolated to develop clonal cell lines expressing either the miR-132 inverse reporter or the reporter control. To determine if the inverse reporter was sensitive to miR-132 expression we constructed a lentivirus and a retrovirus that expresses EGFP and mature miR-132. We tested the level of miR-132 (TaqMan real-time PCR assays) in primary hippocampal cultures infected with the miR-132 expression virus and the miR-132 inverse reporter. The miR-132 expression virus resulted in a 5.5±1.9 fold increase in miR-132 expression (p<0.02 ANOVA with Tukey post-hoc test; n = 2 cultures per condition). The inverse reporter resulted in a slight decrease in miR-132 levels that did not reach statistical significance (0.77±0.22 fold; p>0.9 ANOVA with Tukey post-hoc; n = 2 cultures per condition). Infection of the inverse reporter cell line with the retrovirus overexpressing miR-132 resulted in a loss of mCherry expression in infected cells (Fig. 1c, upper row), but did not alter mCherry expression in the reporter control cell line (Fig. 1c. lower row). These experiments indicate that the inverse reporter can be used as a sensor for miR-132 activity. Furthermore, the inverse reporter has little or no effect on the endogenous levels of miR-132 in hippocampal cultures.

To test the inverse reporter *in vivo*, we co-injected the dentate gyrus of adult mice (6–8 weeks) with equal titers of the inverse reporter or the reporter control, along with a lentivirus constitutively expressing EGFP. The EGFP virus had an identical ubiquitin promoter but lacked the miR-132 target sequences. In contrast to the EGFP virus (FUGW, Fig. 1d, left panel, upper row), the expression of the miR-132 inverse reporter, as detected by mCherry expression (Fig. 1d, center panel, upper row), was limited to cells along the subgranular zone of the dentate gyrus. The reporter control labeled a similar population of cells as the EGFP virus, including cells with the characteristic morphology of mature neurons (Fig. 1d, lower row).

**Figure 2:**
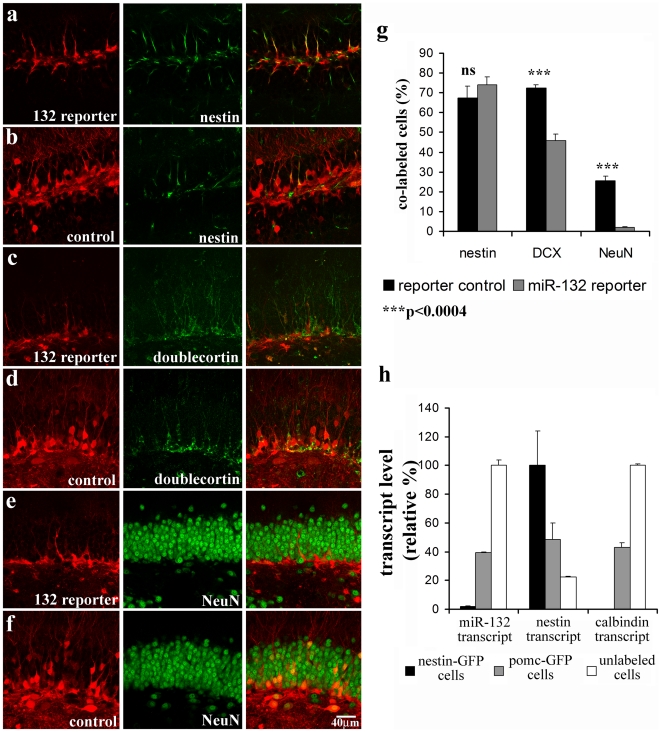
miR-132 Expression Correlated with Maturation of Newborn Granule Neurons in the Adult Dentate Gyrus. An equal titer of lentiviral inverse reporter for miR-132 or the reporter control was injected into the 6 to 8-week-old mouse dentate gyrus and the expression pattern compared at 7 days post-injection. **a,b,** The miR-132 inverse reporter and the inverse reporter control (red) were both highly expressed in nestin immunopositive progenitor cells (green). **c,d,** In the subset of progenitor cells and newborn neurons labeled by doublecortin immunostaining (green), there were fewer doublecortin positive cells expressing the miR-132 inverse reporter than the control. **e,f,** In NeuN immunopositive mature granule cells (green), there was virtually no overlap with expression of the miR-132 inverse reporter (red), whereas the control was expressed in numerous mature granule cells. **g,** The analysis of co-labeled cells showed significant decreases in expression of the inverse reporter in doublecortin (DCX)- and NeuN labeled cells. The data are expressed as a percentage of the immunolabeled-labeled cells that also expressed the inverse reporter or reporter control. A smaller percentage of NeuN than nestin or DCX positive cells were labeled with the control virus, presumably because of the much larger number of mature granule cells. **h,** Using fluorescence activated cell sorting of nestin-GFP or POMC-GFP transgenic animals, we isolated purified populations of precursor cells (black bars; nestin-GFP cells), newborn neurons (gray bars; POMC-GFP cells), and the cell fraction containing mature neurons (white bars; unlabeled cells from POMC-GFP animals). The relative transcript levels of pri-miR-132, nestin, and calbindin were measured using real-time PCR. The miR-132 transcript was highest in mature neurons as supported by the expression of calbindin transcript, whereas the nestin transcript was highest in precursor cells.

To define the cell populations labeled by the inverse reporter, we compared expression of the inverse reporter and the reporter control virus with immunohistochemical markers for precursor cells (nestin), newborn neurons (doublecortin), and mature neurons (NeuN) at 7 days post-injection (Fig. 2). As shown in Figure 2a,b, the majority of nestin-positive precursor cells showed the same level of expression of the inverse reporter as for the reporter control (74.1±8.1% inverse reporter; 67.5±11.9% control), indicating that miR-132 was low or absent in precursor cells. In contrast, the inverse reporter was expressed in a lower percentage of doublecortin positive cells (Fig. 2c,d 45.7±6.7%, inverse reporter; 72.5±3.2%, control), and nearly absent in mature cells immunolabeled with NeuN (2.2±0.5%, inverse reporter; 25.5±4.9%, control; Fig. 2e,f).

We confirmed the pattern of miR-132 expression detected by the inverse reporter by using real-time PCR from cell populations isolated by fluorescence-activated cell sorting (FACS). Precursor cells obtained from nestin-GFP mice [19] showed very low expression of miR-132 whereas newborn neurons from POMC-GFP mice [20], or the cell fraction containing mature granule neurons (non-fluorescent cells sorted from the dentate gyrus of POMC-GFP animals) had increasing levels of miR-132 (Fig. 2h, left). As expected, nestin transcripts showed a reciprocal pattern with high expression in the precursor cell population. Transcript levels of calbindin, a marker of mature granule cells, increased in parallel with miR-132. Thus the immunohistochemical and real-time PCR profiling validate the pattern defined by the inverse reporter, indicating that miR-132 expression increases as granule neurons differentiate and mature.

### miR-132 knockdown impairs functional integration

**Figure 3:**
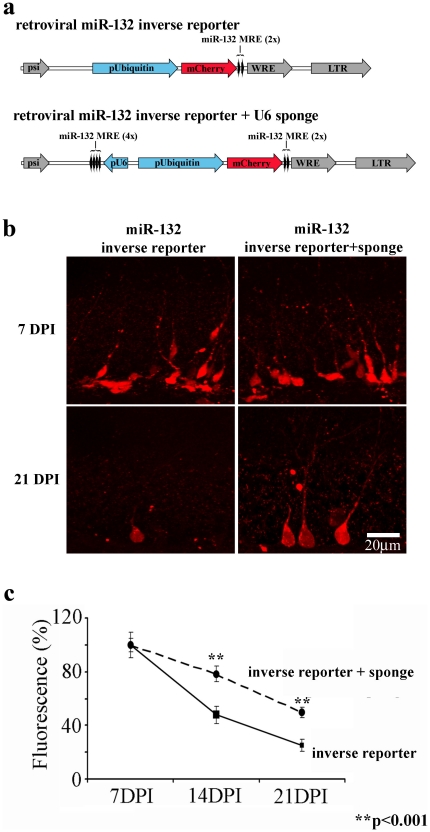
Retroviral Sponge Knockdown of miR-132 Activity *In Vivo*. **a,** The inverse reporter cassette was placed in a retroviral vector, and contained two perfect miR-132 target sequences (miR-132 MRE) downstream of mCherry driven by the ubiquitin promoter (pUbiquitin). To make a ‘sponge’ vector for miR knockdown, the retroviral miR-132 inverse reporter+U6 sponge was constructed by adding four additional perfect miR-132 targets downstream of the U6 promoter. **b,** At 7 DPI (top row), the inverse reporter and the inverse reporter+sponge showed similar expression levels in newborn granule cells *in vivo*. By 21 DPI (bottom row), mCherry showed higher expression in cells expressing inverse reporter+sponge compared to inverse reporter alone, consistent with lower levels of miR-132 in sponge-expressing cells. As indicated by the representative panel in Figure 3b there were also more mCherry+ cells in sponge-injected mice. **c,** We quantified the fluorescence intensity for cells labeled by the inverse reporter+sponge or the inverse reporter as a percentage of the fluorescence at 7 DPI. The fluorescence intensity of cells expressing the inverse reporter+sponge was greater than that of the inverse reporter alone for mice injected 14 and 21 days prior (p<.001, ANOVA with Tukey post-hoc). The increased fluorescence indicates the ability of the sponge cassette to knockdown miR-132 activity relieving repression of the inverse reporter cassette. The number of cells measured was 7 DPI (n = 55, sponge; n = 48, inverse reporter); 14DPI (n = 34, sponge; n = 40, inverse reporter); and 21DPI (n = 51, sponge; n = 35, inverse reporter).

The results above indicate that miR-132 is expressed at the right time and in the right place to influence newborn neurons as they integrate into the adult hippocampal circuitry. To test the functional role of miR-132, we designed a retrovirus to knockdown its expression. Retroviruses only integrate into dividing neurons, which allows targeted genetic manipulation of newborn neurons. We cloned four perfect miR-132 target sites downstream of the U6 promoter to sequester endogenous miR-132, and placed this cassette into the pRubi (Retrovirus with internal ubiquitin promoter; see methods) retroviral vector. To determine the effectiveness of this U6 “sponge” *in vivo*, we co-expressed it with the miR-132 inverse reporter (Fig. 3a). At 7 days post-injection, the expression of the sponge did not alter the fluorescence of the inverse reporter, consistent with the low levels of miR-132 in newborn neurons at this stage (Fig. 3b, top panels). At 14 and 21 days post-injection as endogenous miR-132 increased, cells expressing only the inverse reporter showed a decreased fluorescence compared to 7 days post-injection (Fig. 3b, bottom left panel). However, cells expressing the sponge showed significantly greater fluorescence at 14 and 21 days post-injection (Fig. 3b, bottom right panel), indicating that the sponge reduced endogenous miR-132. At 21 days, the per cell fluorescence was 60.7±5.6% for cells expressing the sponge compared to 25.1±4.3% for cells expressing only the inverse reporter (Fig. 3c).

**Figure 4:**
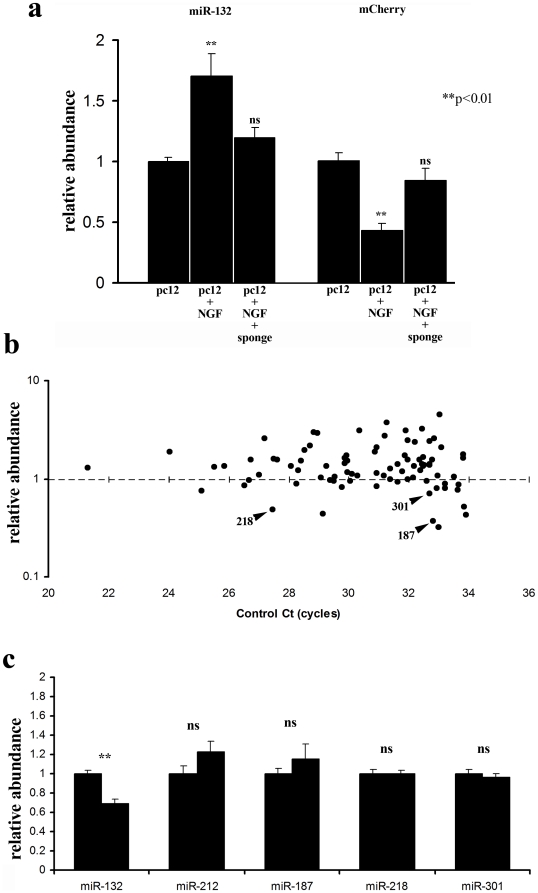
miR-132 Sponge Efficacy and Specificity. To test the efficacy of the sponge we examined whether it altered miR-132 expression in rat PC12 cells. **a,** Treatment of PC12 cells with NGF resulted in a 1.7 fold increase in mature miR-132 expression (p<.001, Newman-Keul test). Infection with the miR-132 sponge prevented this miR-132 induction. To determine the effect of miR-132 induction on a target, we measured mCherry expression in a monoclonal PC12 cell-line expressing the miR-132 inverse reporter. As expected, NGF treatment significantly reduced mCherry expression and this reduction was blocked by the miR-132 sponge. **b,** To test the specificity of the sponge we examined the microRNA expression profile of HEK293 cells infected with the miR-132 sponge. We selected HEK293 cells because they have a very low abundance of miR-132, thus limiting possible indirect effects on other microRNAs as a result of miR-132 knockdown. Using the TaqMan multiplex array we robustly detected 86 microRNAs (Ct<34). Of these, only seven were reduced by more than 25% in the sponge-expressing cells. We cross-referenced these seven microRNAs to microarray data examining microRNAs expressed in the adult dentate gyrus (Luikart, unpublished data). Only three microRNAs were both reduced in the multiplex array and expressed endogenously in the dentate gyrus – miR-187, miR-218, and miR-301. A single multiplex array can yield highly variable results. We therefore assessed whether these potential off-target effects could be validated using larger sample sizes (n = 3 independent experiments) and real-time PCR. **c,** Infection of PC12 cells with the miR-132 sponge resulted in a 32% reduction in miR-132 expression, but did not reduce the expression of miRs-187, 218, 301, or 212 (p>0.3; ANOVA with Tukey post-hoc). miR-212 is highly homologous to miR-132 and generated from the same transcript, making it a particularly good specificity control.

To confirm the efficacy and specificity of the sponge, we measured endogenous miR-132 in PC12 cells using real-time PCR (Fig. 4a). The sponge prevented the NGF-induced increase in miR-132 levels, and prevented the NGF-induced downregulation of the inverse reporter (Fig. 4a). To test for off-target effects of the sponge on other microRNAs, we determined the microRNA expression profile of HEK293 cells infected with the miR-132 sponge. HEK293 cells have a low abundance of miR-132, thus limiting the possibility of indirect effects due to miR-132 knockdown. In a TaqMan multiplex array of HEK293 cells, 86 microRNAs were robustly detected. We focused on 7 of the 86 that were decreased by more than 25% in an array from sponge-treated cells. Of these possible nonspecific targets, only 3 are expressed in adult dentate gyrus -miR-187, miR-218, and miR-301 (Fig. 4b). However, real time PCR did not confirm these miRs as non-specific targets (Fig. 4c). Further, miR-212, which has a similar sequence to miR-132, was not reduced. These results indicate that the sponge is highly specific for miR-132.

**Figure 5:**
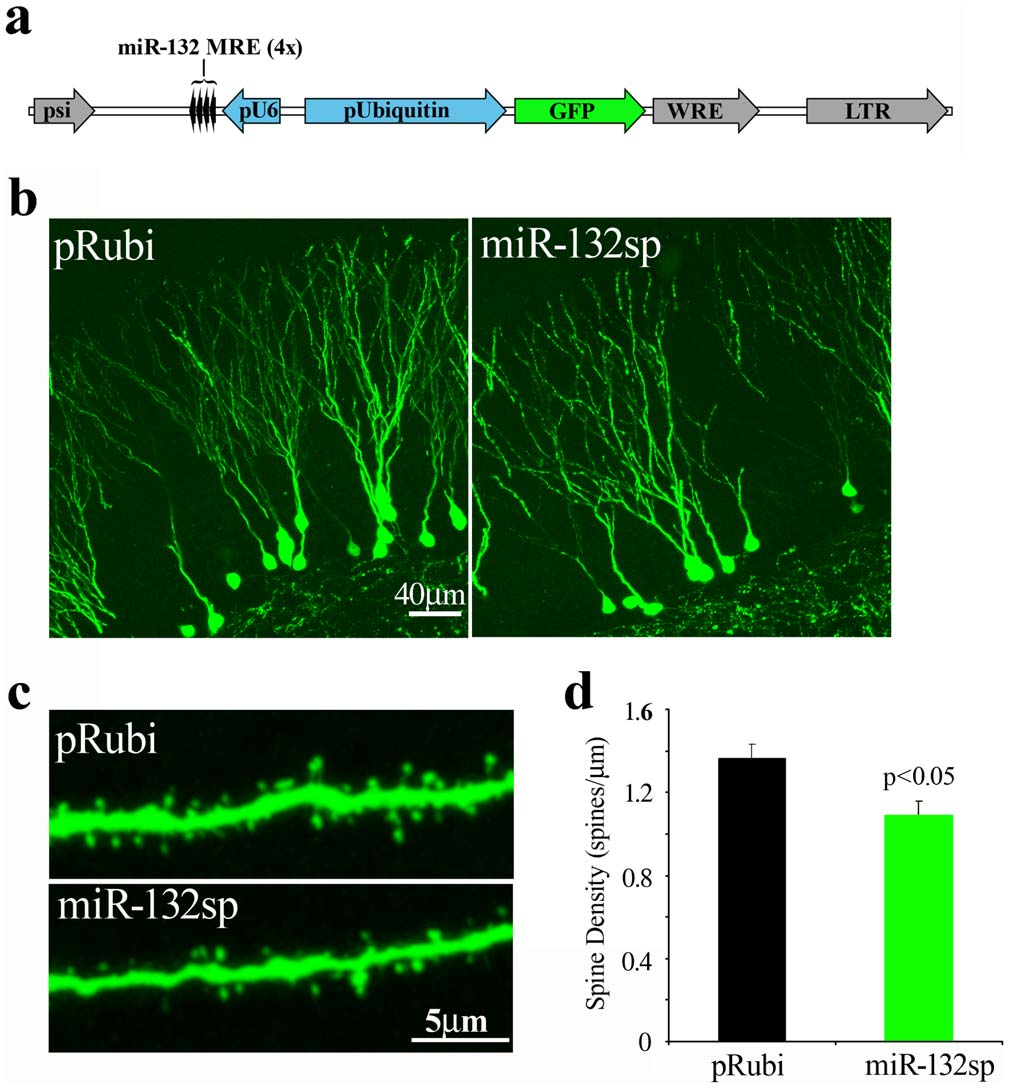
miR-132 Knockdown Decreases Dendritic Spine Density *In Vivo*. **a,** We assessed the effects of miR-132 knockdown on the dendrites of newborn neurons using a retroviral miR-132 sponge (miR-132sp) with four perfect miR-132 targets downstream of the U6 promoter and with EGFP downstream of the ubiquitin promoter. **b,** At 21 days post-injection, dendritic morphology was not altered in cells expressing miR-132sp as compared to cells expressing EGFP alone (pRubi, see text for details). **c,** We compared dendritic spine density in segments from the outer molecular layer in control (pRubi) expressing cells compared to cells expressing the sponge. **d,** Dendritic spine density decreased in cells expressing the sponge (green) compared to the control (black) (dendritic segments from n = 3 mice, control; 24 dendritic segments from n = 4 mice, miR-132sp; p = .004, t test). Analysis of the variances among dendritic segments showed that there was no difference between animals for control or sponge mice (Levene's test, p = .42).

We used the sponge to examine the effect of reducing miR-132 levels on dendrite outgrowth, spine formation and synaptic activity of newborn neurons. A retrovirus expressing the miR-132 sponge and EGFP (Fig. 5a) resulted in brightly labeled cells suitable for electrophysiological and morphological analysis. We chose to focus our analysis on 21-day-post-injections neurons because newborn neurons at this time point have just begun integration into the glutamatergic circuitry, thus allowing for sensitive detection of alterations in synapse or circuit formation. Neurons infected with sponge had no overt differences in somatic size or dendritic morphology compared to cells infected with the pRubi control retrovirus (Fig. 5b). Total dendritic arborization in the pRubi control neurons was 1176±73 µm compared to 1191±50 µm in neurons expressing the sponge (n.s. t-test; 16 neurons and 18 neurons from 3 animals respectively). However, dendritic spine density decreased 21% in cells expressing the sponge (Fig. 5c,d; 1.36±0.06 spines/µm in control, 1.07±0.09 spines/µm in sponge-expressing neurons).

**Figure 6:**
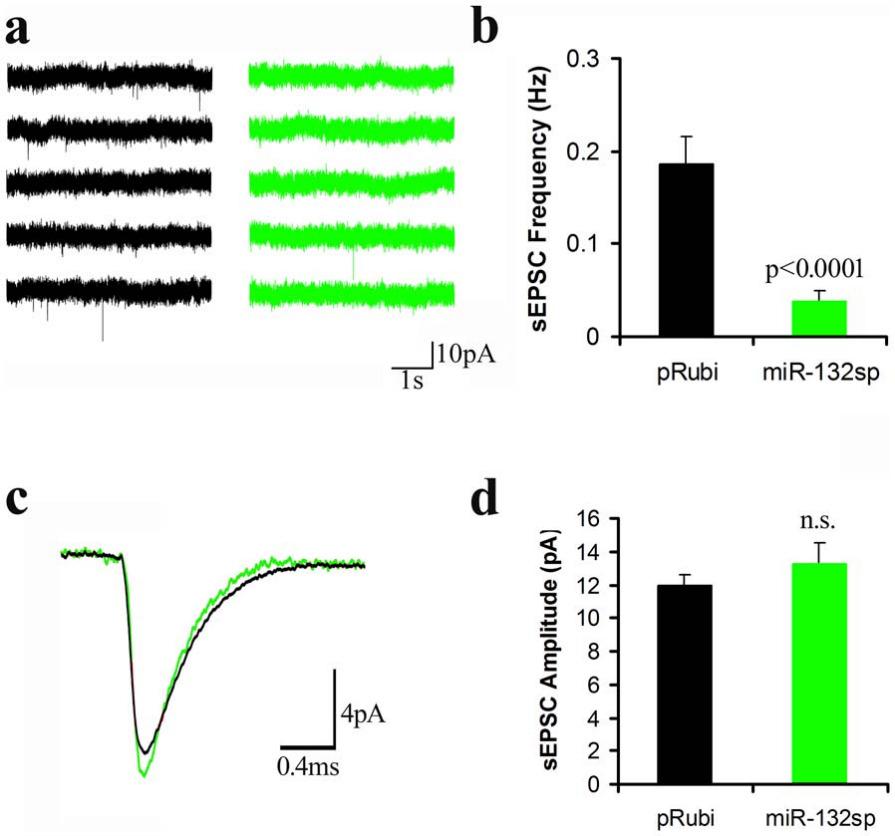
miR-132 Knockdown Markedly Decreased sEPSC Frequency. **a,** Acute brain slices were prepared from mice at 21 DPI. Spontaneous excitatory postsynaptic currents (sEPSCs) were recorded in voltage-clamped (Vh = −70 mV) granule cells labeled with a retrovirus expressing mCherry at 21DPI (control, black trace) or in granule cells expressing miR-132 sponge and GFP (green trace). **b,** At 21 days post-injection, sEPSCs were nearly absent in sponge-infected cells (green), whereas control cells showed the normal low level of sEPSCs expected for newborn neurons. **c,d,** Unscaled average traces from pRubi control (black) and miR-132sp cells (green) revealed no change in sEPSC amplitude. All p-values were calculated using the Kruskal-Wallis test with Conover post-hoc test (n = 16 control cells and n = 12 miR-132sp cells).

To evaluate the impact of miR-132 knockdown on synaptic activity, we prepared acute brain slices from animals infected with either the pRubi control or the miR-132 sponge virus. Synaptic function was assayed at 21 days post-injection using whole-cell recording. During the first 2 weeks post-neurogenesis, newborn neurons receive exclusively GABAergic input before glutamatergic synapses develop during the 3^rd^ week. We monitored the onset of excitatory synaptic activity by measuring the frequency of spontaneous EPSCs. At 21 days post-injection, sEPSCs were extremely rare in neurons expressing the sponge (Fig. 6a,b; 0.19±0.03 Hz, control; 0.04±0.01 Hz, sponge), but there was no significant difference in sEPSC amplitude (Fig. 6c,d). There was no apparent change in the rise time of sEPSCs, although the decay times were somewhat faster in neurons expressing the sponge (4.00±0.25 ms control vs. 2.88±0.23 ms miR-132sp). We did not examine the kinetics further because of the extremely low number of events in sponge-expressing neurons. The input resistance of control neurons was 964±158 mΩ compared to 1485±429 mΩ in neurons expressing the sponge (n.s., Kruskal Wallis test, Conover post-hoc test).

**Figure 7:**
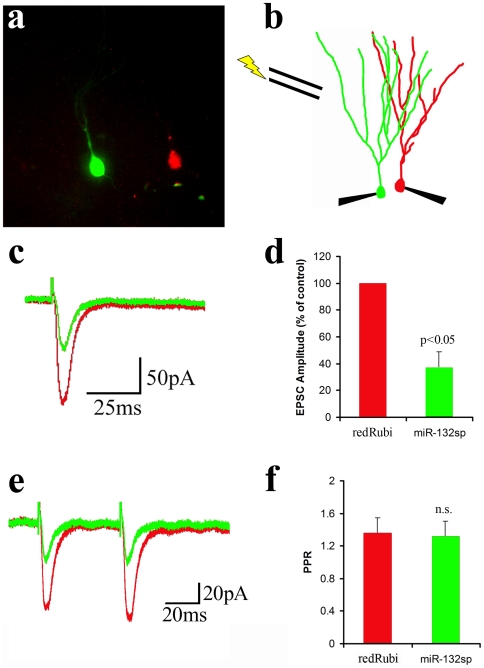
In Paired Recording, Evoked AMPA Currents Were Smaller in miR-132 Knockdown Granule Cells. **a,** We injected equal titers of the mCherry expressing control retrovirus (redRubi) and the EGFP expressing miR-132 sponge retrovirus (miR-132sp) *in vivo*, which resulted in the labeling of control granule cells (red) situated in close proximity to miR-132 knockdown cells (green), as shown for a brain slice on the electrophysiology set-up. **b,** To activate common inputs to both cells, we placed a stimulating electrode in the perforant path and made simultaneous recordings from control (red) and sponge-expressing cells (green). **c,d,** The evoked EPSC was much smaller in granule cells expressing the sponge (green) compared to control (red). **e,f,** The paired pulse ratio (PPR) did not differ between control and sponge (green) cells (p = .82 , paired t-test, n = 4 pairs). All recordings were at −70 mV in solution containing 1 mM Mg^2+^ and SR-95531.

The decrease in spine density combined with the marked decrease in spontaneous EPSCs in sponge-expressing neurons is consistent with a reduction in postsynaptic sites. However, sEPSC frequency can be highly variable and is influenced by presynaptic mechanisms. Our results were also necessarily obtained by comparing neurons in different groups of mice. Thus to examine the synaptic phenotype of miR-132 knockdown more directly, we co-injected a mCherry-expressing control retrovirus (redRubi) and the EGFP-expressing miR-132 sponge virus. This configuration allowed us to identify control cells (red) and miR-132 knockdown cells (green) that have the same birthdate in a single animal (Fig. 7a). We then stimulated the perforant path and made simultaneous recordings from control neurons and neighboring miR-132 sponge-expressing neurons (Fig. 7b). In neurons expressing the miR-132 sponge, the evoked EPSC was much smaller than in the control cell (63.2±11.9% reduction, n = 4, Fig. 7c,d), but there was no change in the paired-pulse ratio (PPR; Fig. 7e,f, 1.36±0.19, control; 1.32±0.19, sponge). The lack of change in the PPR indicates that the release probability of perforant pathway axons onto control and sponge-expressing cells was unaltered. Thus the decrease in the evoked EPSC amplitude suggests that there are fewer synapses or fewer AMPA receptors at individual synapses in miR-132 knockdown cells. Taken together, the decreases in spine density, sEPSC frequency, and evoked EPSC amplitude indicate that miR-132 knockdown resulted in decreased synapse formation on newborn neurons.

### miR-132 knockdown increases “inflammatory” signaling

**Figure 8:**
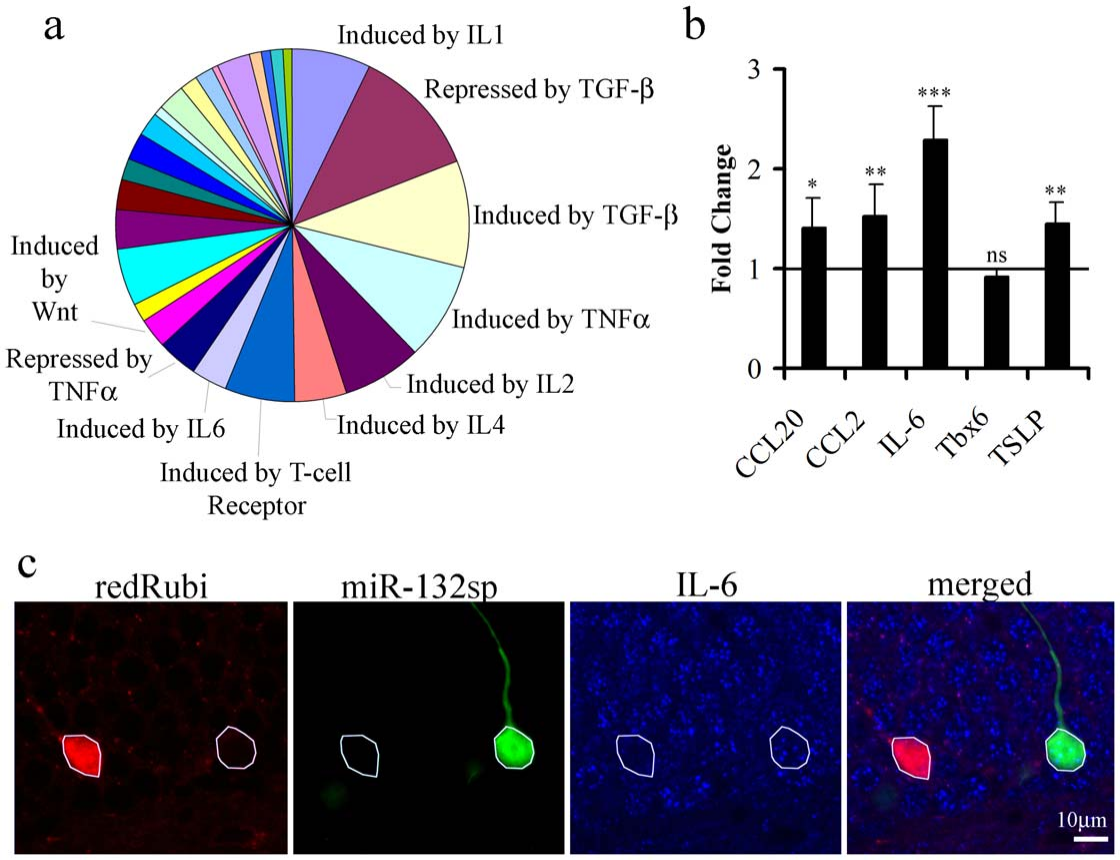
miR-132 Knockdown Correlates to Increased Expression of Molecules Regulated by Immune Signaling Pathways. miR-132 was knocked down in PC12 cells infected with the miR-132 sponge virus (Figure 4). Whole rat genome microarrays were probed to screen for changes in miR-132 knockdown cells vs. control-infected cells. **a,** Functional grouping of genes that increased when miR-132 was knocked down indicated a significant enrichment in genes regulated by signaling pathways that are involved in immune function. The pie chart represents all categories for the upregulated genes. The labeled categories are the 8 most significantly enriched. **b,** Using real-time PCR we confirmed an increase in 4 out of 5 genes that were upregulated on the microarray. **c,** At 21 days post-injection miR-132 sponge expressing neurons (green) had an increase in IL-6 immunofluorescence (compare blue in white outlined areas in 3rd panel from the left) as compared to control redRubi infected neurons (red), consistent with the result for IL-6 in the microarray.

To determine the ensemble of genes affected by knockdown of miR-132 we performed a microarray analysis in PC12 (ATCC) cells expressing the miR-132 sponge. RNA isolated from NGF-treated PC12 cells infected either with the miR-132 sponge or an EGFP control virus were hybridized to Agilent whole rat genome microarrays. In three independent experiments, 309 genes were consistently upregulated and 224 were consistently downregulated as compared to cells infected with the EGFP control (ArrayExpress accession E-MTAB-458). To see if there was a pattern to the transcriptional changes elicited by miR-132 knockdown, we focused on the set of upregulated genes that would be expected to include genes directly targeted by miR-132. We functionally grouped upregulated genes by the cell signaling pathways that regulate their expression (Fig. 8a). There was significant enrichment in genes regulated by 10 signaling pathways. Of these, 7 of the identified pathways have classical inflammatory or immune functions (IL-1, TNFα, IL-2, IL-4, T-cell receptor, and IL-6). Using real-time PCR we validated the genes that were identified on the microarray. In a subset of 5 genes, we confirmed enrichments in 4: interleukin-6 (IL-6), chemokine ligand 2 (MCP-1/CCL2), chemokine ligand 20 (CCL20), and thymic stromal lymphopoietin (TSLP) (Fig. 8b).

To determine whether genes identified in PC12 cells also were regulated by miR-132 knockdown *in vivo*, we used IL-6 as a test case because of its involvement in nervous system function [21]. Immunostaining confirmed robust expression of IL-6 in granule neurons of the adult dentate gyrus. Consistent with previous reports the level of IL-6 was higher in mature granule neurons than in the immature granule neurons labeled with retrovirus. Further, IL-6 expression was increased at 21 days post-infection for granule neurons expressing the miR-132 sponge compared to control neurons expressing only mCherry (Fig. 8c). The fluorescence intensity of neurons expressing the miR-132 sponge was 2.93±0.63 compared to 0.23±0.94 for control (p<0.05; t-test; n = 24 and 20 cells, respectively). Thus for IL-6, changes in protein expression in granule cells *in vivo* paralleled the RNA changes in PC12 cells.

## Discussion

We took advantage of the characteristic developmental sequence of newborn granule cells in the dentate gyrus to assess the role of the activity-dependent microRNA, miR-132 *in vivo*. The pattern of miR-132 expression we observed is consistent with a role for miR-132 in the maturation of neurons during development as well as for newborn neurons in the adult. The timing of expression, and the impact of knockdown on excitatory synapse formation, indicates that miR-132 coordinates an instructive transcriptional program that is necessary for the integration of newborn neurons into the adult central nervous system. Our microarray analysis of miR-132 knockdown suggests a link to inflammatory genes in this program.

### Monitoring and manipulating miR levels in vivo

Mature microRNAs work by blocking translation or causing degradation of their target mRNAs. Thus to detect endogenous levels of miR-132 *in vivo*, we designed an inverse reporter whose expression was blocked by miR-132 activity. Several microRNA reporter strategies, based on cloning of a perfect microRNA target sequence into the 3′ UTR of a marker have been described. This approach was first used to monitor expression in transgenic models [22], [23], [24]. In hematopoetic cells Brown et al., incorporated microRNA target sequences into lentiviral vectors to achieve cell-type specific expression based on known microRNA expression patterns [25]. Our results show that this approach can be adapted to examine the expression pattern of a microRNA *in vivo*. Using lenti- and retroviral delivery systems, we were able to show not only the pattern, but also the timing of expression of miR-132 in newborn neurons in the adult brain. Further, the inverse reporter, which had 2 MREs driven by ubiquitin had little or no effect on endogenous miR-132 levels and had no effect on dendritic spine density (data not shown).

MicroRNAs present special challenges in designing loss of function experiments because multiple microRNAs can be expressed from the same transcript. 2′ O-methyl oligonucleotides are typically used to inhibit single microRNAs *in vitro* using standard transfection methods. MicroRNA “sponges” are emerging as a genetically encodable method to knockdown specific microRNAs [26], [27]. This approach has been successfully adapted to lentiviral mediated genetic manipulation in the circulatory system *in vivo* [28], but has not been previously reported for the CNS. By using a retroviral vector for delivery, we were able to target newborn neurons. The knockdown in our hands was efficacious in that the sponge doubled the expression of the inverse reporter, but it did not restore the inverse reporter to the levels seen in cells lacking miR-132. The incomplete knockdown by the sponge presumably reflects an imbalance between the endogenous levels of miR-132 and the number of targets introduced [28]. Multiplex miR arrays allowed us to screen for miRs that might cause off-target effects. Of the miRs identified in the array, no off-target effects were confirmed with real-time PCR. Our results indicate that viral-mediated knockdown of miRs *in vivo* can be efficient and highly specific.

### The role of miR-132 in activity-dependent development of newborn neurons

Newborn neurons are a useful model system for studies of activity dependent phenomena *in vivo* because of their stereotypical developmental stages. Neuronal activity increases the number of adult-generated neurons and enhances their integration. Although the molecular mechanisms underlying this process are still being identified, neuronal activity causes transcriptional changes that drive the elaboration of the mature neuronal phenotype [7]. In the dentate gyrus, newborn granule cells are depolarized primarily by GABAergic input within two weeks of their terminal division [29]. Early GABAergic depolarization is essential to the normal integration of newborn neurons *in vivo* [30], and results in a transient increase in CREB activity and c-fos transcription [8], [12], [31]. CREB activation likely contributes to processes such as growth, synapse formation, and synapse strengthening by regulating the expression of many genes [13].

Several microRNAs have been identified as putative CREB targets including miR-132 [13]. Our results show that miR-132 levels increase just as newborn neurons begin to receive neuronal activity. Likewise, pilocarpine-induced seizures also cause a rapid increase in miR-132 (data not shown; [32]), as well as an associated increase in dendritic outgrowth and synapse formation *in vivo* [11]. Increases in BDNF mRNA expression parallel the seizure-induced increase in miR-132 (data not shown). BDNF is also a CREB target that contributes to neuronal differentiation, growth, and synapse formation [33], [34], [35]. Although the sequence in this molecular cascade remains to be elucidated, the evidence supports the notion that miR-132 contributes to the activity- and CREB-dependent development of adult-generated neurons.

### The synaptic phenotype of miR-132

Knockdown of miR-132 activity *in vivo* caused a decrease in dendritic spine density, sEPSC frequency, and evoked EPSCs in newborn neurons. At the time of recording, 21 days post-injection, normal newborn neurons have elaborated dendrites into the outer molecular layer and have formed spiny synapses with perforant path inputs [36]. We did not see a change in total dendritic arborization, indicating that the effect was primarily on synapse formation or maturation. Previous studies have used the 2′ O-methyl technique to knockdown miR-132 *in vitro*. In dissociated cultures, early knockdown (3–7 DIV) resulted in decreased neurite outgrowth and spine density [16], [17]. However, in organotypic slice cultures, the dendritic length and spine density of CA1 pyramidal neurons was unaffected under basal conditions, but the inhibitor did block the increase in dendritic growth and spine density triggered by increasing neuronal activity with a 48 hour bicuculline treatment [16], [18]. A recent report indicates that inhibition of miR-132 with a ‘sponge’ had little effect on dendritic spines in cultured neurons [37]. However, this sponge was introduced into more mature (14 DIV) neurons.

The differences in various preparations emphasize the importance of using *in vivo* approaches for loss of function studies. Our results indicate that miR-132 is expressed at basal levels of neuronal activity *in vivo*. Retroviral-mediated miR-132 sponge expression attenuated the endogenous onset of miR-132 expression during adult neurogenesis, and markedly decreased dendritic spine density and functional synaptic activity. It is likely that a complete knockout of miR-132 would have a more severe effect on newborn neurons.

### miRs and their targets

A single microRNA can affect the expression of hundreds of genes [14], [15]. For example, one form of inheritable progressive hearing loss results from a mutation in the miR-96 seed region, which alters the expression of a large array of targets both directly and indirectly [38], [39], indicating that the phenotype reflects the concerted action of many target genes. Similarly, miR-31 suppresses breast cancer metastasis via a number of putative targets [40]. These authors took advantage of their model system in which cells could be genetically manipulated *in vitro* and then transplanted back into the animal for *in vivo* analysis. No single target could rescue the phenotype *in vivo*, but concomitant knockdown of three targets resulted in rescue [41]. Unfortunately, this elegant approach is not yet feasible for neurons *in vivo*.

In our microarray analysis of PC12 cells, knockdown of miR-132 results in the upregulation of 335 and downregulation of 224 genes. Several miR-132 targets have been explored as possible mediators of a neuronal phenotype. For example, overexpression of the miR-132 target, GTPase activating protein (p250GAP), mimicked the *in vitro* effects of miR-132 knockdown on spine formation and synapse function [16], [17], [18]. Another miR-132 target, MeCP2, has been postulated to form a regulatory feedback system guiding synaptic maturation [42]. We did not detect changes in the expression of these genes at the RNA level in PC12 cells. However, the previous reports indicate that they are regulated through translational repression and changes can only be detected at the protein level. By examining RNA levels, genes regulated by signaling pathways involved in inflammation and immunity were among the most enriched. In non-neuronal cells miR-132 is an important regulator of immune signaling [43], [44]. However many inflammatory and immune molecules are also expressed in neurons and may have non-immune functions in the CNS [45]. For example, immune molecules have been implicated in normal developmental synaptic pruning and inhibition of synapse formation following neuronal injury [45]. Further, previous studies have reported that immune signaling negatively impacts neurogenesis [46], [47]. Our data suggest that immune signaling may be linked to the subsequent steps whereby newborn neurons integrate into the synaptic circuitry of the adult.

We do not identify direct targets of miR-132, but characterize the overall effect that miR-132 knockdown produces at the cellular and molecular level. An exhaustive identification of direct miR-132 targets will improve our understanding of the mechanisms by which miR-132 regulates neuronal function. Newborn neurons are a good cell type to explore the ensemble of gene products regulated by miR-132 during synapse formation. These cells have a well-defined sequence of development and relatively pure populations can be isolated for bioinformatic studies. However, such studies await improvements in the efficiency of isolating and characterizing the proteome of newborn neurons - and in the ability to knockdown arrays of pleotropically functioning genes in individual neurons.

## Methods

All animal procedures were performed as approved by the institutional animal care and use committee (IACUC) and followed NIH guidelines for the ethical treatment of animals (IACUC ID IS00000455). For harvesting of brain tissue (see specific protocols below), mice were deeply anesthetized and euthanized by decapitation.

### Cloning

#### The miR-132 inverse reporter

GFP was excised from the FUGW lentiviral vector [48] using BamHI/EcoRI. mCherry was cloned from pRSETB using the BamHI/EcoRI sites [49]. Two perfect miR-132 target sites with 100% homology to the mature miR sequence (indicated by capital letters below) or their reverse complement were then cloned into the EcoRI site downstream of mCherry by annealing and ligating the following oligos:

5′ aattc CGACCATGGCTGTAGACTGTTA ggcgcgcc CGACCATGGCTGTAGACTGTTA g

5′ aattc TAACAGTCTACAGCCATGGTCG ggcgcgcc TAACAGTCTACAGCCATGGTG g

The top sequence was used to construct the inverse reporter and the reverse complement sequence was used as the reporter control as shown in Figure 1.

#### pRubi (Retrovirus with internal ubiquitin promoter) and redRubi

A BstBI site was introduced into pSie [30] by ligating a linker into the BamHI/XbaI digested vector. pSie and FUGW were then digested with XhoI and BstBI to isolate the retroviral backbone from pSie and the ubiquitin promoter, GFP, and WRE fragment from FUGW. These fragments were then ligated to generate the pRubi retroviral construct. GFP was then replaced with mCherry using the EcoRI/BamHI sites in pRubi to generate redRubi.

#### pCMVU6 3.0

To make pCMVU6 compatible with unique restriction sites in both pRubi as well as FUGW a PacI linker was ligated into the NheI site.

### Fluorescent-activated cell sorting (FACS)

To isolate purified populations of precursor cells or newborn neurons, hippocampi were isolated from brain tissue of nestin-GFP mice (Yu et al., 2005) and dentate gyrus of POMC-GFP mice (Overstreet et al., 2004), respectively. Tissue pieces were isolated under a fluorescence microscope in dissection media ((NaCl (137 mM); KCl (5.3 mM); HEPES (10 mM); D-glucose (33 mM); sucrose (44 mM); NaHPO_4_•7H_2_O (0.167 mM); KH_2_PO_4_ (0.220 mM); phenol red (0.067 mM); 1% pen/strep), then digested with papain for 40 minutes (10 ml D1; 2 mg cysteine; 150 ul calcium solution (100 uM); 100 ul EDTA solution (50 mM); 200 units papain). The digestion was stopped with 10 ml heat inactivated fetal calf serum, 25 mg bovine serum albumin and 25 mg trypsin inhibitor, and tissue was triturated to a single cell suspension. RNAse inhibitor (200 units/5 ml media) was added to the dissociation media for cell sorting. The flow cytometer (FACS Vantage Diva, BD Biosciences, San Jose, CA) had a 488 nm, 200 mW argon-ion laser equipped with a 530/30 nm bandpass filter for EGFP, and was operated with the following settings: 10 psi, 20,000 drops/s, 130 µm tip. The nestin-GFP mouse was engineering such that expression is limited to neuronal precursors and not epithelial cells (Yu et al., 2005). The unlabeled cell fraction for the POMC-GFP animals largely contains the mature granule cell population and was used as the “mature neuron” sample. Cells collected from the cell sorter were used to isolate RNA, generate cDNA and perform QPCR.

### QPCR

Hippocampal cultures were prepared from P0 mice as previously described [50]. Cultures were infected with lentiviral particles on the day of plating with 10 particles per neuron and RNA isolated at 7 days *in vitro*. PC12 cells and HEK293 cells were obtained from ATCCRNA was isolated using trizol (Invitrogen) and the manufacturers recommended procedures. The samples were DNAse treated using the DNAfree kit (Ambion) followed by an additional ethanol precipitation. For traditional transcripts and pri-miRs cDNAs were generated from 200 ng of RNA using random hexamer primers of the First Strand cDNA Synthesis Kit (Fermentas). One sample was serially diluted to provide a standard curve for the real time reaction. All other samples were diluted 1∶3. The real time reaction was performed in duplicate using FastStart SYBR Green Master (Roche). Quantification of mature microRNAs was performed using the TaqMan MicroRNA Assay with 100 ng of input RNA (Applied Biosystems). One sample was serially diluted to generate the standard curve and all others diluted 1∶2. PCR was performed on an Opticon OP346 (MJ Research). A exhaustive list of QPCR primer sequences can be provided on request.

### Microarray Experiments

Microarray experiments were performed by Miltenyi Biotechnology using standard procedures. All data is MIAME compliant and the raw data has been deposited in the MIAME compliant database ArrayExpress (accession E-MTAB-458). Experiments were performed in triplicate, amplifying 1 µg of total rat RNA with the Agilent Quick Amp Labeling Kit (Agilent Technologies). 825 ng of Cy3 labeled control (redRubi infected) and Cy5 labeled experimental (miR-132 sponge infected) cRNA were combined and hybridized overnight to Agilent whole rat genome microarrays. Fluorescence signals were detected using the microarray scanner. Microarray images were analyzed using the Agilent feature extraction software and Rosetta Resolver (Rosetta Biosoftware) to determine fold changes and statistical confidence. Upregulated genes with an average p<0.05 from all three arrays were functionally grouped by molecular pathway. Inclusion of a gene as induced by or repressed by a particular pathway was defined by pathway curations from Miltneyi biotechnology and Netpath. Fisher's exact test and the Benjamini Hochberg correction were applied to determine the significance of enrichment of a category.

### Histology

Mice were deeply anesthetized with 2% avertin injected IP, and were intracardially perfused with ice-cold PBS+4% sucrose, followed by 4% (w/v) paraformaldehyde (PFA) in PBS+4% sucrose. The brain was postfixed in 4% PFA+4% sucrose overnight and embedded in 2.5% agarose. 50 µm or 100 µm thick coronal sections were cut using a vibratome (Leica). For immunohistochemistry, slices were incubated with mouse anti-nestin (1∶50, Chemicon MAB353), mouse anti-NeuN (1∶500, Chemicon MAB377), guinea pig anti-doublecortin (1∶500, Chemicon Ab5910), rabbit anti-IL-6 (1∶500, Abcam ab6672) and Alexa488 conjugated anti-GFP (Molecular Probes). Primary antibodies were detected using secondary antibodies conjugated with Cy3 (Jackson Immunoresearch, West Grove, PA) followed by counterstaining with DAPI (Vector Labs). IL-6 immunofluorescence was measured using Image J and manually circling GFP positive miR-132 sponge expressing neurons or mCherry positive control neurons. To correct for variability in overall staining intensity the background fluorescence intensity of the entire image was subtracted from the intensity of the individual cell of interest.

### Stereotaxic Injections

Mice were anesthetized using an isoflurane gas system (Veterinary Anesthesia Systems Co.). The mouse was placed in a Kopf stereotaxic frame fitted with a gas nose cone - a skin incision was made and holes were drilled at x: ±1.1 mm, y: −1.9 mm from bregma. Using a 10 µl Hamilton syringe with a 30 ga needle and the Quintessential Stereotaxic Injector (Stoelting), 1 l of virus was delivered at 0.25 µl/min at z-depths of 2.5 and 2.3 mm. The syringe was left in place for 1 min after each injection before being slowly withdrawn. The skin above the injection site was closed using veterinary glue. Animals receive post-operative lidocaine and drinking water containing children's Tylenol.

### Electrophysiology

Anesthetized adult mice were perfused with an ice-cold solution containing (in mM): 110 choline Cl, 7 MgCl_2_, 2.5 KCl, 1.25 NaH_2_PO_4_*2H_2_O, 0.5 CaCl_2_, 1.3 Na-ascorbate, 25 NaHCO_3_ bubbled with 95% O_2_-5% CO_2_. After decapitation, hippocampi were harvested and sliced in the same solution. Slices were stored and recordings performed in a solution containing (in mM): 125 NaCl, 25 NaHCO_3_, 2.5 KCl, 1.25 NaH_2_PO_4_, 2.0 CaCl_2_, 1.0 MgCl_2_, and 25 D-glucose, bubbled with 95% O_2_-5% CO_2_. For EPSC recordings, we added SR95531 (10 µM) to block GABA_A_ receptors, and we used a Csgluconate pipette solution (in mM): 100 gluconic acid, 0.2 EGTA, 5 HEPES, 2 Mg-ATP, 0.3 Li-GTP, pH = 7.2, 295mOsm (pH to 7.2 using 50%CsOH for a final concentration of 100–120 Csgluconate). Series resistance (5–20 MΩ) was monitored, and experiments were discarded if the resistance increased by >10 MΩ. Currents were filtered at 4 kHz and sampled at 40 kHz (MultiClamp 700A Molecular Devices, Union City, CA). Synaptic responses were evoked with a tungsten bipolar electrode in the outer molecular layer of the dentate gyrus. Paired pulse ratios were calculated as the ratio of peak amplitudes of 10 averaged episodes. The inter-stimulus interval was 50 ms. Spontaneous synaptic currents were detected using template matching in Axograph X. The template rise time was 1 ms, decay time 6 ms, baseline 10 ms, length 30 ms and a threshold of three. Captured events were then manually reviewed. One-way ANOVA statistics were performed in Igor Pro, paired t-tests in Microsoft Excel, and Kolmogorov-Smirnov test or the Kruskal-Wallis test in BrightStat [51]. All values are reported as mean ± SEM.

### Morphology

For all morphological analyses, image z-stacks were obtained using a Zeiss LSM 510 laser scanning confocal microscope. Spine density was estimated from image stacks captured within 100 µm from the tips of the dendrites in the outer molecular layer. Images were captured using a 63× Plan-Apochromat oil lens (Zeiss) at 512×512 and an electronic zoom of 3×. The virtual section thickness was 0.8 µm with a step size of 0.5 µm and a scan speed of 9. Using Image J, a dendritic section was traced by hand and each spine head was manually circled to measure dendritic spine density. No correction was made for spines obscured in the z-plane.

### Statistics

Statistical comparisons used t-tests or ANOVA for multiple comparisons unless otherwise indicated. Significance was set at p<.05. For data presented in the figures, the specific tests used are indicated in the figure legends. Statistical tests for data included only in the text are indicated in the text.

## References

[1] Zhao C, Deng W, Gage FH (2008). Mechanisms and functional implications of adult neurogenesis.. Cell.

[2] Sahay A, Drew MR, Hen R (2007). Dentate gyrus neurogenesis and depression.. Prog Brain Res.

[3] Li Y, Luikart BW, Birnbaum S, Chen J, Kwon CH (2008). TrkB regulates hippocampal neurogenesis and governs sensitivity to antidepressive treatment.. Neuron.

[4] Santarelli L, Saxe M, Gross C, Surget A, Battaglia F (2003). Requirement of hippocampal neurogenesis for the behavioral effects of antidepressants.. Science.

[5] van Praag H, Kempermann G, Gage FH (2000). Neural consequences of environmental enrichment.. Nat Rev Neurosci.

[6] Zhao CS, Overstreet-Wadiche L (2008). Integration of adult generated neurons during epileptogenesis.. Epilepsia.

[7] Flavell SW, Greenberg ME (2008). Signaling mechanisms linking neuronal activity to gene expression and plasticity of the nervous system.. Annu Rev Neurosci.

[8] Overstreet-Wadiche LS, Bensen AL, Westbrook GL (2006). Delayed development of adult-generated granule cells in dentate gyrus.. J Neurosci.

[9] Fujioka T, Fujioka A, Duman RS (2004). Activation of cAMP signaling facilitates the morphological maturation of newborn neurons in adult hippocampus.. J Neurosci.

[10] Lee B, Dziema H, Lee KH, Choi YS, Obrietan K (2007). CRE-mediated transcription and COX-2 expression in the pilocarpine model of status epilepticus.. Neurobiol Dis.

[11] Overstreet-Wadiche LS, Bromberg DA, Bensen AL, Westbrook GL (2006). Seizures accelerate functional integration of adult-generated granule cells.. J Neurosci.

[12] Jagasia R, Steib K, Englberger E, Herold S, Faus-Kessler T (2009). GABA-cAMP response element-binding protein signaling regulates maturation and survival of newly generated neurons in the adult hippocampus.. J Neurosci.

[13] Impey S, McCorkle SR, Cha-Molstad H, Dwyer JM, Yochum GS (2004). Defining the CREB regulon: a genome-wide analysis of transcription factor regulatory regions.. Cell.

[14] Selbach M, Schwanhausser B, Thierfelder N, Fang Z, Khanin R (2008). Widespread changes in protein synthesis induced by microRNAs.. Nature.

[15] Baek D, Villen J, Shin C, Camargo FD, Gygi SP (2008). The impact of microRNAs on protein output.. Nature.

[16] Impey S, Davare M, Lasiek A, Fortin D, Ando H (2010). An activity-induced microRNA controls dendritic spine formation by regulating Rac1-PAK signaling.. Mol Cell Neurosci.

[17] Vo N, Klein ME, Varlamova O, Keller DM, Yamamoto T (2005). A cAMP-response element binding protein-induced microRNA regulates neuronal morphogenesis.. Proc Natl Acad Sci U S A.

[18] Wayman GA, Davare M, Ando H, Fortin D, Varlamova O (2008). An activity-regulated microRNA controls dendritic plasticity by down-regulating p250GAP.. Proc Natl Acad Sci U S A.

[19] Yu TS, Dandekar M, Monteggia LM, Parada LF, Kernie SG (2005). Temporally regulated expression of Cre recombinase in neural stem cells.. Genesis.

[20] Overstreet LS, Hentges ST, Bumaschny VF, de Souza FS, Smart JL (2004). A transgenic marker for newly born granule cells in dentate gyrus.. J Neurosci.

[21] Vereyken EJ, Bajova H, Chow S, de Graan PN, Gruol DL (2007). Chronic interleukin-6 alters the level of synaptic proteins in hippocampus in culture and in vivo.. Eur J Neurosci.

[22] Brennecke J, Hipfner DR, Stark A, Russell RB, Cohen SM (2003). bantam encodes a developmentally regulated microRNA that controls cell proliferation and regulates the proapoptotic gene hid in Drosophila.. Cell.

[23] De Pietri Tonelli D, Calegari F, Fei JF, Nomura T, Osumi N (2006). Single-cell detection of microRNAs in developing vertebrate embryos after acute administration of a dual-fluorescence reporter/sensor plasmid.. Biotechniques.

[24] Mansfield JH, Harfe BD, Nissen R, Obenauer J, Srineel J (2004). MicroRNA-responsive ‘sensor’ transgenes uncover Hox-like and other developmentally regulated patterns of vertebrate microRNA expression.. Nat Genet.

[25] Brown BD, Cantore A, Annoni A, Sergi LS, Lombardo A (2007). A microRNA-regulated lentiviral vector mediates stable correction of hemophilia B mice.. Blood.

[26] Care A, Catalucci D, Felicetti F, Bonci D, Addario A (2007). MicroRNA-133 controls cardiac hypertrophy.. Nat Med.

[27] Ebert MS, Neilson JR, Sharp PA (2007). MicroRNA sponges: competitive inhibitors of small RNAs in mammalian cells.. Nat Methods.

[28] Gentner B, Schira G, Giustacchini A, Amendola M, Brown BD (2009). Stable knockdown of microRNA in vivo by lentiviral vectors.. Nat Methods.

[29] Overstreet Wadiche L, Bromberg DA, Bensen AL, Westbrook GL (2005). GABAergic signaling to newborn neurons in dentate gyrus.. J Neurophysiol.

[30] Ge S, Goh EL, Sailor KA, Kitabatake Y, Ming GL (2006). GABA regulates synaptic integration of newly generated neurons in the adult brain.. Nature.

[31] Tashiro A, Makino H, Gage FH (2007). Experience-specific functional modification of the dentate gyrus through adult neurogenesis: a critical period during an immature stage.. J Neurosci.

[32] Nudelman AS, DiRocco DP, Lambert TJ, Garelick MG, Le J (2010). Neuronal activity rapidly induces transcription of the CREB-regulated microRNA-132, in vivo.. Hippocampus.

[33] Luikart BW, Zhang W, Wayman GA, Kwon CH, Westbrook GL (2008). Neurotrophin-dependent dendritic filopodial motility: a convergence on PI3K signaling.. J Neurosci.

[34] Gorski JA, Zeiler SR, Tamowski S, Jones KR (2003). Brain-derived neurotrophic factor is required for the maintenance of cortical dendrites.. J Neurosci.

[35] Bibel M, Barde YA (2000). Neurotrophins: key regulators of cell fate and cell shape in the vertebrate nervous system.. Genes Dev.

[36] Overstreet-Wadiche LS, Westbrook GL (2006). Functional maturation of adult-generated granule cells.. Hippocampus.

[37] Edbauer D, Neilson JR, Foster KA, Wang CF, Seeburg DP (2010). Regulation of synaptic structure and function by FMRP-associated microRNAs miR-125b and miR-132.. Neuron.

[38] Mencia A, Modamio-Hoybjor S, Redshaw N, Morin M, Mayo-Merino F (2009). Mutations in the seed region of human miR-96 are responsible for nonsyndromic progressive hearing loss.. Nat Genet.

[39] Lewis MA, Quint E, Glazier AM, Fuchs H, De Angelis MH (2009). An ENU-induced mutation of miR-96 associated with progressive hearing loss in mice.. Nat Genet.

[40] Valastyan S, Reinhardt F, Benaich N, Calogrias D, Szasz AM (2009). A pleiotropically acting microRNA, miR-31, inhibits breast cancer metastasis.. Cell.

[41] Valastyan S, Benaich N, Chang A, Reinhardt F, Weinberg RA (2009). Concomitant suppression of three target genes can explain the impact of a microRNA on metastasis.. Genes Dev.

[42] Klein ME, Lioy DT, Ma L, Impey S, Mandel G (2007). Homeostatic regulation of MeCP2 expression by a CREB-induced microRNA.. Nat Neurosci.

[43] Lagos D, Pollara G, Henderson S, Gratrix F, Fabani M (2010). miR-132 regulates antiviral innate immunity through suppression of the p300 transcriptional co-activator.. Nat Cell Biol.

[44] Shaked I, Meerson A, Wolf Y, Avni R, Greenberg D (2009). MicroRNA-132 potentiates cholinergic anti-inflammatory signaling by targeting acetylcholinesterase.. Immunity.

[45] Boulanger LM, Shatz CJ (2004). Immune signalling in neural development, synaptic plasticity and disease.. Nat Rev Neurosci.

[46] Carpentier PA, Palmer TD (2009). Immune influence on adult neural stem cell regulation and function.. Neuron.

[47] Monje ML, Toda H, Palmer TD (2003). Inflammatory blockade restores adult hippocampal neurogenesis.. Science.

[48] Lois C, Hong EJ, Pease S, Brown EJ, Baltimore D (2002). Germline transmission and tissue-specific expression of transgenes delivered by lentiviral vectors.. Science.

[49] Shaner NC, Campbell RE, Steinbach PA, Giepmans BN, Palmer AE (2004). Improved monomeric red, orange and yellow fluorescent proteins derived from Discosoma sp. red fluorescent protein.. Nat Biotechnol.

[50] Tovar KR, Westbrook GL (1999). The incorporation of NMDA receptors with a distinct subunit composition at nascent hippocampal synapses in vitro.. J Neurosci.

[51] Stricker D (2008). BrightStat.com: free statistics online.. Comput Methods Programs Biomed.

